# Immunization With a DNA Vaccine Cocktail Encoding TgPF, TgROP16, TgROP18, TgMIC6, and TgCDPK3 Genes Protects Mice Against Chronic Toxoplasmosis

**DOI:** 10.3389/fimmu.2018.01505

**Published:** 2018-06-29

**Authors:** Nian-Zhang Zhang, Qi Gao, Meng Wang, Hany M. Elsheikha, Bo Wang, Jin-Lei Wang, Fu-Kai Zhang, Ling-Ying Hu, Xing-Quan Zhu

**Affiliations:** ^1^State Key Laboratory of Veterinary Etiological Biology, Key Laboratory of Veterinary Parasitology of Gansu Province, Lanzhou Veterinary Research Institute, Chinese Academy of Agricultural Sciences, Lanzhou, China; ^2^Hunan Entry-Exit Inspection and Quarantine Bureau, Changsha, China; ^3^Faculty of Medicine and Health Sciences, School of Veterinary Medicine and Science, University of Nottingham, Loughborough, United Kingdom; ^4^Department of Mathematics, University of Leicester, Leicester, United Kingdom; ^5^Jiangsu Co-Innovation Center for the Prevention and Control of Important Animal Infectious Diseases and Zoonoses, Yangzhou University College of Veterinary Medicine, Yangzhou, China

**Keywords:** *Toxoplasma gondii*, chronic toxoplasmosis, cocktail DNA vaccine, multistage antigens, mixed Th1/Th2 immune response

## Abstract

Toxoplasmosis is a zoonotic disease caused by the intracellular protozoan *Toxoplasma gondii*; and a major source of infection in humans is *via* ingestion of *T. gondii* tissue cysts. Ultimately, the goal of anti-toxoplasmosis vaccines is to elicit a sustainable immune response, capable of preventing formation of the parasite tissue cysts—or, at least, to restrain its growth. In this study, we formulated a cocktail DNA vaccine and investigated its immunologic efficacy as a protection against the establishment of *T. gondii* cysts in the mouse brain. This multicomponent DNA vaccine, encoded the TgPF, TgROP16, TgROP18, TgMIC6, and TgCDPK3 genes, which play key roles in the pathogenesis of *T. gondii* infection. Results showed that mice immunized *via* intramuscular injection three times, at 2-week intervals with this multicomponent DNA vaccine, mounted a strong humoral and cellular immune response, indicated by significantly high levels of total IgG, CD4^+^ and CD8^+^ T lymphocytes, and antigen-specific lymphocyte proliferation when compared with non-immunized mice. Immunization also induced a mixed Th1/Th2 response, with a slightly elevated IgG2a to IgG1 ratio. The increased production of proinflammatory cytokines gamma-interferon, interleukin-2, and interleukin-12 (*p* < 0.0001) correlated with increased expression of p65/RelA and T-bet genes of the NF-κB pathway. However, no significant difference was detected in level of interleukin-4 (*p* > 0.05). The number of brain cysts in immunized mice was significantly less than those in non-immunized mice (643.33 ± 89.63 versus 3,244.33 ± 96.42, *p* < 0.0001), resulting in an 80.22% reduction in the parasite cyst burden. These findings indicate that a multicomponent DNA vaccine, encoding TgPF, TgROP16, TgROP18, TgMIC6, and TgCDPK3 genes, shows promise as an immunization strategy against chronic toxoplasmosis in mice, and calls for a further evaluation in food-producing animals.

## Introduction

*Toxoplasma gondii*, the causative agent of toxoplasmosis, infects nearly all warm-blooded vertebrates, including birds and humans (1, 2). Toxoplasmosis in immunocompetent people usually manifests as a flu-like, self-limiting infection; however, in immunocompromised patients (cancer, AIDS, and organ transplant recipients), reactivation of chronic toxoplasmosis can cause fatal complications, such as toxoplasmic encephalitis (3, 4). The reactivation of tissue cysts is manifested by their rupture, followed by the conversion of released bradyzoites to tachyzoites, and a proliferation of tachyzoites. Therefore, the removal of *T. gondii* tissue cysts from infected individuals would prevent such reactivation. Tissue cysts of *T. gondii* in animals also present a potential threat to human health if they are consumed in raw or undercooked meat (5, 6). Currently, there are no available drugs to eliminate *T. gondii* tissue cysts (7, 8). It is therefore imperative that new approaches are developed for immunotherapy against this infection.

Development of vaccines to prevent *T. gondii* tissue cyst formation can be an effective approach to ensure the safety of meat products, originating from food-producing animals under backyard and free-ranging conditions (9, 10). Over the last two decades, various anti-*T. gondii* vaccine approaches have been evaluated in animal models. Previous studies have shown that DNA vaccine can induce, through enhanced humoral and cellular immune responses, immune protection against acute and chronic toxoplasmosis in animal models (11–15). Additional advantages of the DNA vaccination, when compared with using live-type vaccines, are their thermal stability, safety, and the low cost of production (12, 14). To date, no single DNA vaccine has provided full protection against *T. gondii* cyst formation (9, 16, 17).

*Toxoplasma gondii*-specific cytotoxic T lymphocytes (CTLs) induced by immunization can improve the protective immunity against parasite infection (9, 14). Previous studies have shown that a multicomponent vaccine may offer better protection than a single antigen (16–19) due to the elevated numbers of *T. gondii*-specific CTLs, and the subsequent increase in the production of antigen-specific cytokine gamma-interferon (IFN-γ) (19, 20). A combination of different antigens, in theory, contain more CTL epitopes and are considered superior to a single antigen for protecting the host against *T. gondii* infection (14, 21).

This study aimed to examine the protective efficacy of a DNA multicomponent vaccine against chronic *T. gondii* infection. Five well-characterized antigens play key roles in host–parasite interaction including host cell attachment (MIC6) (22), gliding motility and invasion (profilin) (23, 24), signal transduction and egress (calcium-dependent protein kinase 3; CDPK3) (25), intracellular proliferation (ROP18) (26), and modulation of host gene expression (ROP16) (27). These antigens were selected to formulate a cocktail DNA vaccine. We investigated the immunologic efficacy of this vaccine, in protecting Kunming mice against chronic *T. gondii* infection. In addition, we conducted a longitudinal immune analysis and evaluated several immunization strategies, in order to provide some guidance for optimal schedules of vaccine administration in future clinical trials. Utilization of the DNA vaccine with multi-antigens is a step forward in the development of commercial vaccine formulations against chronic toxoplasmosis for use in humans and food-producing animals.

## Materials and Methods

### Ethics Statement

Animal experiments were reviewed and approved by the Animal Administration and Ethics Committee of Lanzhou Veterinary Research Institute, Chinese Academy of Agricultural Sciences. The study was performed in strict compliance with the recommendations set forth in the Animal Ethics Procedures and Guidelines of the People’s Republic of China. All efforts were made to minimize animal suffering and to reduce the numbers of animals used in the experiments.

### Mice and Parasite Strain

Specific pathogen-free female Kunming mice, aged 6–8 weeks, were purchased from the Center of Laboratory Animals, Lanzhou Institute of Biological Products, Lanzhou, China. They were housed, in pathogen-free conditions, at Lanzhou Veterinary Research Institute in controlled room under stable conditions (12-h/12-h dark/light cycle, 50–60% humidity, and ~22°C temperature). Mice had access to sterilized food and water *ad libitum* and were acclimated for 1 week before use. The avirulent *T. gondii* type II Prugniuad (Pru) was propagated in our laboratory, by oral passage of infected brain homogenates in Kunming mice (28). Bradyzoites of *T. gondii* Pru strain were used to prepare the *T. gondii* lysate antigen (TLA) as described previously (29, 30).

### Preparation of Multicomponent DNA Vaccine

The pVAX1 plasmids encoding *T. gondii* profilin (pVAX1-PF), rhoptry protein 16 (pVAX1-ROP16), rhoptry protein ROP18 (pVAX1-ROP18), microneme protein 6 (pVAX1-MIC6), and calcium-dependent protein kinase 3 (pVAX1-CDPK3) were constructed as previously reported (31–35), with the fidelity of all plasmids confirmed by sequencing (Sangon, China). The five eukaryotic plasmids were each transformed into *E. coli* DH5α for propagation and were isolated by anion exchange chromatography (EndoFree Plasmid Giga Kit, Qiagen Sciences, MD, USA) following the manufacturer’s instruction. Plasmid concentration and purity was determined by measuring the optical density ratio *A*_260_/*A*_280_. The purified plasmids were stored at −20°C until used in the mouse immunization protocols.

### Immunization and Challenge

Mice (*n* = 120) were randomly allocated to six groups of 20 mice. Mice in groups G1, G2, and G3 were immunized using plasmids encoding either five genes, four genes, or one gene, respectively. Further details of the various vaccination regimens are listed in Table 1. Mice received intramuscular (i.m.) injections of 100 µg of plasmids in 100 µl phosphate-buffered saline (PBS), into the tibialis anterior muscles using a 1-ml insulin syringe with a 28-G needle. Three vaccinations at 2-week intervals were performed. Mice in G4 and G5 received empty pVAX1 vector and PBS, respectively. Mice in G6 were healthy untreated controls (non-immunized and uninfected). Following the primary immunization, mice in G1–G5 were provided with two booster immunizations at weeks 2 and 4. For the challenge, 2 weeks after the final immunization (6 weeks post initial immunization), 6 mice from each group were inoculated orally with 200 µl of PBS containing 10 tissue cysts of the avirulent *T. gondii* Pru strain. Control mice received 200 µl of PBS without cysts. Six weeks post challenge, the mouse brains were removed, homogenized in 1 ml of PBS and cysts were morphologically identified and counted under a microscope (40× objective) on three aliquots of 20 µl, without staining.

**Table 1:** Vaccination regimens used in this study.

Group	Immunization protocol	Content	Volume	Administration
G1	pVAX1 plasmids expressing ROP16 + ROP18 + MIC6 + CDPK3 + PF	20 μg/plasmid	100 µl	Intramuscular
G2	pVAX1 plasmids expressing ROP16 + ROP18 + MIC6 + CDPK3	25 μg/plasmid	100 µl	Intramuscular
G3	pVAX1-PF	100 µg	100 µl	Intramuscular
G4	pVAX1	100 µg	100 µl	Intramuscular
G5	Phosphate-buffered saline	–	100 µl	Intramuscular
G6	Healthy control	–	–	–

### Multicomponent DNA Vaccine Induced a Systemic Humoral Immune Response

The blood samples from three mice in each group were collected from the tail vein pre-immunization, and 2 weeks after each of the three sequential immunizations (i.e., at 2, 4, and 6 weeks post immunization). The sera were separated by centrifugation of blood samples at 3,000 × *g* for 10 min. Levels of anti-*T. gondii*, total IgG, and IgG isotypes (IgG2a and IgG1 antibodies, as markers for Th1 and Th2 responses) were examined in mice in each group using the SBA Clonotyping System-HRP Kit according to the manufacture’s instruction (Southern Biotech Co., Ltd., Birmingham, AL, USA). Wells of 96-well microtiter plates were coated with 100 µl (10 µg/ml) TLA diluted in PBS at 4°C overnight, and then washed with PBST (PBS with 0.05% Tween-20). Plates were then treated with PBS-T plus 1% low fat milk for 1 h at ambient temperature, in order to block non-specific binding sites. After washing the wells three times with PBS, mouse serum samples (1:10 diluted with PBS) were added to the wells, and incubated at 37°C for 1 h followed by washing three times with PBST. The serum from non-immunized mice was used as a negative control. Horse radish peroxidase (HRP) conjugated anti-mouse IgG (1:500 diluted with PBS) and anti-mouse IgG1 or IgG2a (1:1,000 diluted with PBS) were added to the wells and incubated for 1 h at 37°C. Wells were then washed five times with PBST, and streptavidin–horseradish peroxidase was added for 1 h at ambient temperature. TMB (3,3′,5,5′-tetramethyl benzidine) in 200 µl citrate-phosphate buffer (0.05 M Na_2_HPO_4_, 0.025 M citric acid, pH 4.0) and 2 mM H_2_O_2_ were added to monitor the peroxidase activity. The reaction was stopped after 30 min by adding 2 M H_2_SO_4_. Analysis of antibody responses was based on absorbance, measured at 450 nm using an ELISA plate reader (iMark microplate absorbance reader; Bio-Rad, Hercules, CA, USA). All measurements were performed in triplicate.

### Lymphocyte Proliferation Assay

Spleens from five mice in each group were aseptically collected, 2 weeks after the final/third booster immunization and were pushed through a fine nylon mesh. After removal of red blood cells, using erythrocyte lysis buffer (Sangon, China), the purified splenocytes were re-suspended in RPMI medium, supplemented with 10% fetal calf serum and 100 U/ml penicillin/streptomycin. The number of purified splenic lymphocytes was determined, and cells were cultured at a concentration of 2 × 10^5^ cells/well in 96-well flat-bottom microwell plates, in complete RPMI medium. Cell cultures were stimulated with TLA (10 or 5 µg/ml) in three wells. RPMI media only (no antigen) and Concanavalin A (ConA; 5 µg/ml; Sigma, St. Louis, MO, USA) were used as nonstimulated and positive controls, respectively. After 4 days at 37°C in a humidified 5% CO_2_ incubator, the level of *in vitro* proliferative response was determined using the 3-(4,5-dimethylthiazol-2-yl)-5-(3-carboxymethoxyphenyl)-2-(4-sulfophenyl)-*2H*-tetrazolium, inner salt (MTS) assay (Promega, USA). The OD values were measured using a microplate reader (iMark microplate absorbance reader; Bio-Rad, Hercules, CA, USA) at 490 nm. Data were expressed as stimulation index (SI), which was calculated as the ratio of mean OD_590_ values in immunized and control groups.

### Antigen-Specific T-Cell Proliferation

The percentages of CD4^+^ and CD8^+^ T lymphocytes in the purified splenocytes were determined by flow cytometry. The specific antigen epitope of each T subclass was stained with phycoerythrin-labeled anti-mouse CD3 (eBioscience), allophycocyanin-labeled anti-mouse CD4 (eBioscience), and fluorescein isothiocyanate-labeled anti-mouse CD8 (eBioscience) antibodies. The cell suspension was then fixed with FACScan buffer (PBS containing 1% BSA and 0.1% sodium azide) and 2% paraformaldehyde. All samples were analyzed for their fluorescence profiles on a FACScan flow cytometer (BD Biosciences) using System II software (Coulter).

### Cytokines

Splenocytes at a density of 2 × 10^5^ cells/well were co-cultured with TLA and medium only (negative control). Culture supernatants were harvested at 24 h for quantification of interleukin-2 (IL-2) and IL-4 and at 96 h for IFN-γ and interleukin-12 (IL-12). The level of each cytokine was determined using commercial ELISA kits, according to the manufacturer’s instructions (BioLegend, USA). The supernatants from each cell culture were pipetted into microplate wells followed by assay buffer A. Then, 100 µl of the detection solution was added into each well. After washing the plate four times, avidin-HRP A solution and the substrate solution E were added sequentially. The reaction was stopped by adding 100 µl of stop solution. The sensitivity limits for the assays were 4 pg/ml for IL-12 (p70), 20 pg/ml for IFN-γ, 10 pg/ml for IL-4, and 50 pg/ml for IL-2. Optical absorbance was read at 450 nm. This experiment was performed in triplicate.

We studied the expression of the two transcription factors, p65/RelA and T-bet of the NF-κB pathway, in an effort to establish their roles in mediating the increased production of T cell cytokine (e.g., IFN-γ and IL-12). Total RNA from 10^7^ purified splenocytes of mice from G1 to G6 were extracted using Trizol reagent (Invitrogen, USA), as per the manufacturer’s instructions. RNAs were dissolved in RNase-free ddH_2_O (TaKaRa, China) and reverse transcribed first-strand cDNAs were used as templates for real-time (RT)-PCR. The primers for amplification of RelA/p65 and T-bet genes are listed in Table 2. β-Actin was used as a housekeeping reference gene. The SYBR Green qPCR SuperMix was purchased from Invitrogen (USA). RT-PCR was performed on ABI PRISM^®^ 7500 Sequence Detection System (Applied Biosystems). The amplification reactions were performed under the following conditions: 50°C 2 min, 95°C 2 min, 40 cycles of 95°C for 15 s, and 60°C for 32 s. Melting curve analysis was carried out under the following conditions: 1 min at 95°C, 65°C for 2 min, and progressive increase from 65 to 95°C to ensure that a single product was amplified in each reaction. All measurements were run in triplicate.

**Table 2:** Sequences of primers used for amplification of p65/RelA, T-bet, and β-actin genes.

Primer name	Sequence
T-bet-F	5′-GCCAGGGAACCGCTTATATG-3′
T-bet-R	5′-TGGAGAGACTGCAGGACGAT-3′
RelA-F	5′-GAACCAGGGTGTGTCCATGT-3′
RelA-R	5′-TCCGCAATGGAGGAGAAGTC-3′
β-Actin-F	5′-GCTTCTAGGCGGACTGTTAC-3′
β-Actin-R	5′-CCATGCCAATGTTGTCTCTT-3′

### Statistical Analysis

Two-way ANOVA with matched data at different weeks was used to compare the total IgG antibody responses between the mouse groups. Tukey’s multiple comparisons test was then employed to test the differences between each of the three vaccination groups, and each of the three control groups at each week. One-way ANOVA and Tukey’s multiple comparisons test were used for comparison in regards to the levels of IgG1 and IgG2a, and IgG2a/IgG1 ratio, proliferation of splenocytes, the numbers of CD3^+^ CD4^+^ CD8^+^ and CD3^+^ CD8^+^ CD4^+^ T cells, cytokine production, and the numbers of brain cysts. Welch’s *t* test was used to compare the qPCR in the three genes (p65/RelA, T-bet, and β-actin) between the blank control group and mice in group 1. Data are presented as mean ± SD. All analyses and graphs were performed using GraphPad Prism version 7.04 (San Diego, CA, USA). The level of significance was defined as *p ≤ 0.05, ** *p* ≤ 0.01, ***p ≤ 0.001, and ****p ≤ 0.0001.

## Results

### Identification of Plasmids

Five purified plasmids pVAX1-PF, pVAX1-ROP16, pVAX1-ROP18, pVAX1-MIC6, and pVAX1-CDPK3 were confirmed by sequencing prior to use in the immunization experiment. Sequence alignment analysis showed that no base deletion or change was detected after alignment with the corresponding sequences in GenBank: accession numbers AY937257.1, DQ116422, AM075204, EF102772, and AJ488146.2.

### Kinetics of Humoral Immune Responses

**Figure 1:**
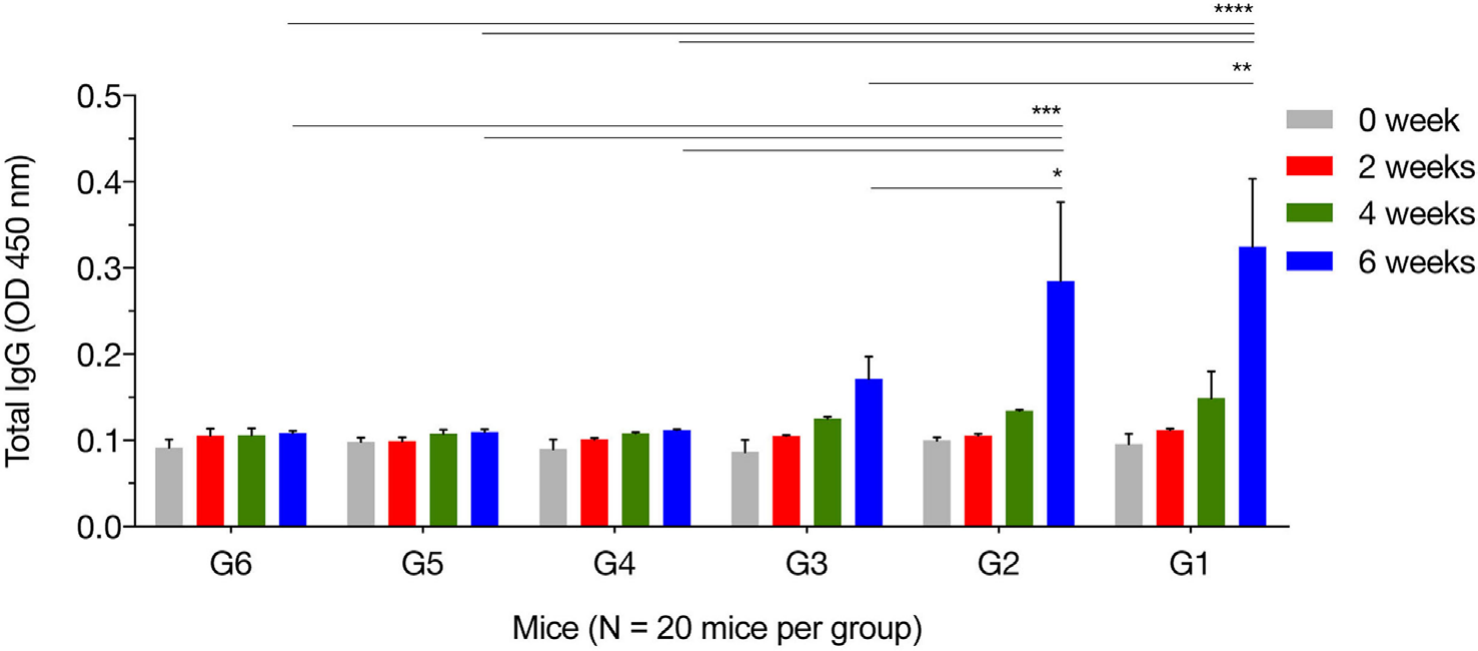
Antigen-specific antibody response in immunized mice. Total IgG antibodies were determined in the sera of mice pre-immunization (0 week) and at 2, 4, and 6 weeks post three consecutive booster immunizations. Experimental groups include G1 mice received pVAX1 plasmids containing five antigens (ROP16, ROP18, MIC6, CDPK3, and PF); G2 mice received pVAX1 plasmids containing four antigens (ROP16, ROP18, MIC6, and CDPK3); G3 mice received pVAX1-PF; G4 mice received an empty pVAX1; G5 mice received phosphate-buffered saline alone; G6 healthy control mice. Data are mean ± SDs for three wells (representative of three independent experiments). *p < 0.05, **p < 0.01, ***p < 0.001, and ****p < 0.0001, compared with the control groups.

We characterized temporal changes in the humoral immune responses following immunization. Sera were available pretreatment, and at weeks 2, 4, and 6 post sequential immunizations from mice in all groups. Levels of total IgG and its subclasses (IgG2a and IgG1) from these time points were determined using ELISA. As shown in Figure 1, the IgG responses tended to increase with the increased number of immunizations, and with the increased number of plasmids used in immunization. Levels of IgG in mice in G1, G2, and G3 increased proportionally with time following immunization, and peaked at 2 weeks after the third/final booster immunization. Statistical analyses of the results (for weeks 0, 2, 4, and 6 post immunization) were performed using a two-way ANOVA for matched data. Both time and group variables had significant effects *(p* < 0.0001 for time and *p* = 0.0140 for group). Tukey’s multiple comparisons test showed no significant differences in all groups at week 0, 2, and 4 after immunization. At week 6 post immunization, the levels of IgG production in mice from G1 were significantly higher than those in the control groups G4, G5, and G6 (*p* < 0.0001, *p* < 0.0001, and *p* < 0.0001, respectively). The levels of IgG in G2 mice were significantly elevated compared with the control groups G4, G5, and G6 (*p* = 0.0004, *p* = 0.0004, and *p* = 0.0003, respectively). The levels of IgG in G3 mice were significantly high compared with the control groups G4, G5, and G6 (*p* = 0.6142, *p* = 0.5803, and *p* = 0.5576, respectively). The levels of IgG antibodies in mice from control groups (G4, G5, and G6) were not significantly different when compared with each other. Within G1, G2, and G3 groups, levels of IgG antibodies were not significantly different in G1 versus G2 (*p* = 0.8977), but were significantly different in G1 versus G3 (*p* = 0.0023) and in G2 versus G3 (*p* = 0.0447).

**Figure 2:**
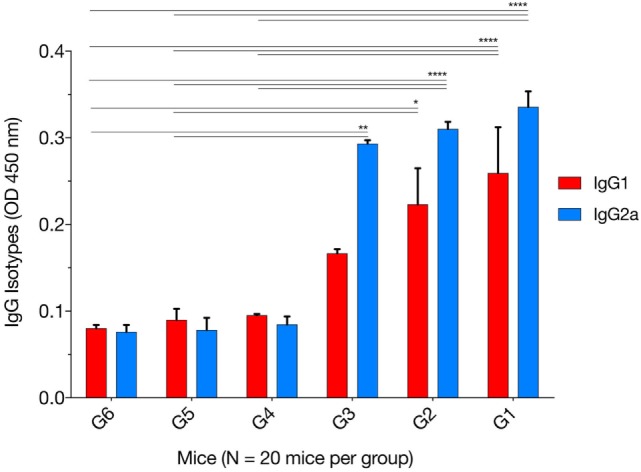
Levels of IgG subclasses (IgG1 and IgG2a) in the sera of mice 2 weeks after the final booster immunization. The patterns of IgG2a to IgG1 post immunization using various immunization regimens suggest the induction of a mixed Th1/Th2 immune response. Experimental groups include G1: mice received pVAX1 plasmids containing five antigens (ROP16, ROP18, MIC6, CDPK3, and PF); G2: mice received pVAX1 plasmids containing four antigens (ROP16, ROP18, MIC6, and CDPK3); G3: mice received pVAX1-PF; G4: mice received an empty pVAX1; G5: mice received phosphate-buffered saline alone; G6: healthy control mice. Each bar represents the group OD_450_ ± SDs for three wells (representative of three experiments). *p < 0.05, **p < 0.01, and ****p < 0.0001, compared with the control groups.

The levels of IgG1 and IgG2a were determined 2 weeks after the final immunization. One-way ANOVA and Tukey’s multiple comparisons test were used for statistical analysis of IgG1 and IgG2a data. The results of IgG1 analysis showed that G1 versus G4, G5, and G6 were all significantly different; G2 versus G4 (*p* = 0.0626), G2 versus G5 (*p* = 0.0504), G2 versus G6 (*p* = 0.0335), whereas G3 versus G4, G5, and G6 were not significant (Figure 2). The levels of IgG2a showed that G1 versus G4, G5, and G6 were all significant; G2 versus G4, G5, and G6 were all significant; and G3 versus G4 (*p* = 0.0765), G3 versus G5 (*p* = 0.0053), and G3 versus G6 (*p* = 0.0049). These results suggest that both IgG1 and IgG2a antibodies were higher in mice in G1, G2, and G3 than those in control groups at 2 weeks after the third immunization, suggesting a mixed Th1/Th2 immune response. To provide information on the dominant cellular immune type (Th1 or Th2) induced by immunization, ELISA was used to determine the ratio of IgG2a to IgG1 in the sera of all mouse groups. Results showed that immunized mouse groups, in particular, mice immunized with pVAX1-PF, had a slightly Th1-biased immune response as indicated by the higher IgG2a/IgG1 ratio (G1: 1.29; G2: 1.38; G3: 1.75) when compared with that of the control mouse groups (G4: 0.88; G5: 0.87; G6: 0.94; *p* = 0.0877).

### Cellular Immune Responses

**Figure 3:**
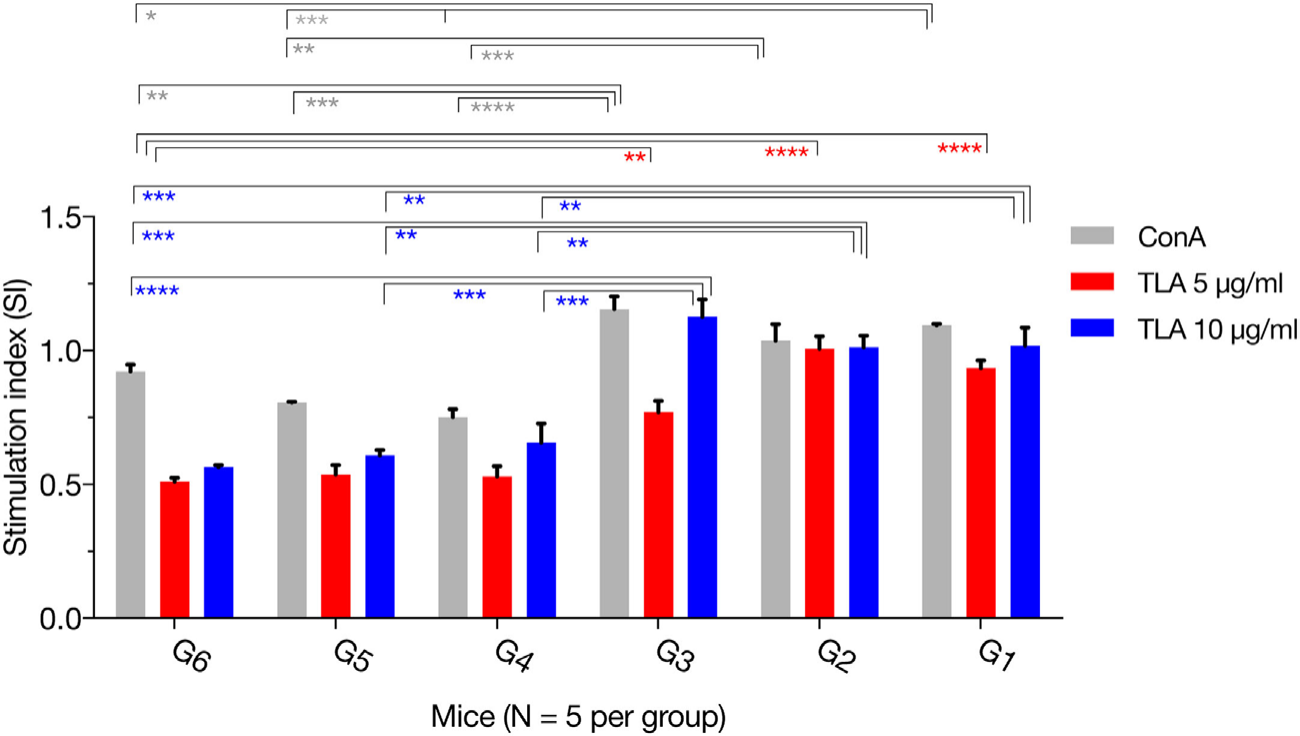
*In vitro* lymphocyte proliferation induced by immunization. Splenocyte proliferation was assessed in mice 2 weeks after the final booster immunization. Exposure of splenocytes to 10 or 5 µg/ml *T. gondii* lysate antigen (TLA) significantly increased T cell proliferation in splenocytes obtained from mice in G1, G2, and G3, compared with proliferation in the control using antigen alone suggesting the induction of antigen-specific T-cell immune response after DNA immunization. Experimental groups include G1: mice received pVAX1 plasmids containing five antigens (ROP16, ROP18, MIC6, CDPK3, and PF); G2: mice received pVAX1 plasmids containing four antigens (ROP16, ROP18, MIC6, and CDPK3); G3: mice received pVAX1-PF; G4: mice received an empty pVAX1; G5: mice received phosphate-buffered saline alone; G6: healthy control mice. Each sample was assayed at least in triplicate. Data represent the mean ± SD (error bars) from the five mice. *p < 0.05, **p < 0.01, ***p < 0.001, and ****p < 0.0001, compared with the control groups.

The MTS assay was used to assess the T lymphocyte’s proliferative response following stimulation with TLA or ConA. As expected, there was no difference for the SIs between the immunized groups (G1, G2, and G3). However, *in vitro* lymphocyte proliferation assay revealed that splenic lymphocytes from immunized mice had a significantly higher SI than their counterparts from non-immunized controls, either in the presence of ConA or TLA extract. Exposure to 10 µg/ml TLA increased T-cell proliferation in G1, G2, and G3 compared with that obtained from control mice (Figure 3): G1 versus G6 (*p* = 0.0010), G2 versus G6 (*p* = 0.0012), and G3 versus G6 (*p* = 0.0001). Similar results for T lymphocyte proliferation were obtained from splenocytes sensitized with 5 µg/ml TLA in mice from G1, G2, and G3 (Figure 3). The results of this *ex vivo* splenic lymphocyte proliferation assay suggested that immunization has induced antigen-specific lymphocyte proliferation.

**Figure 4:**
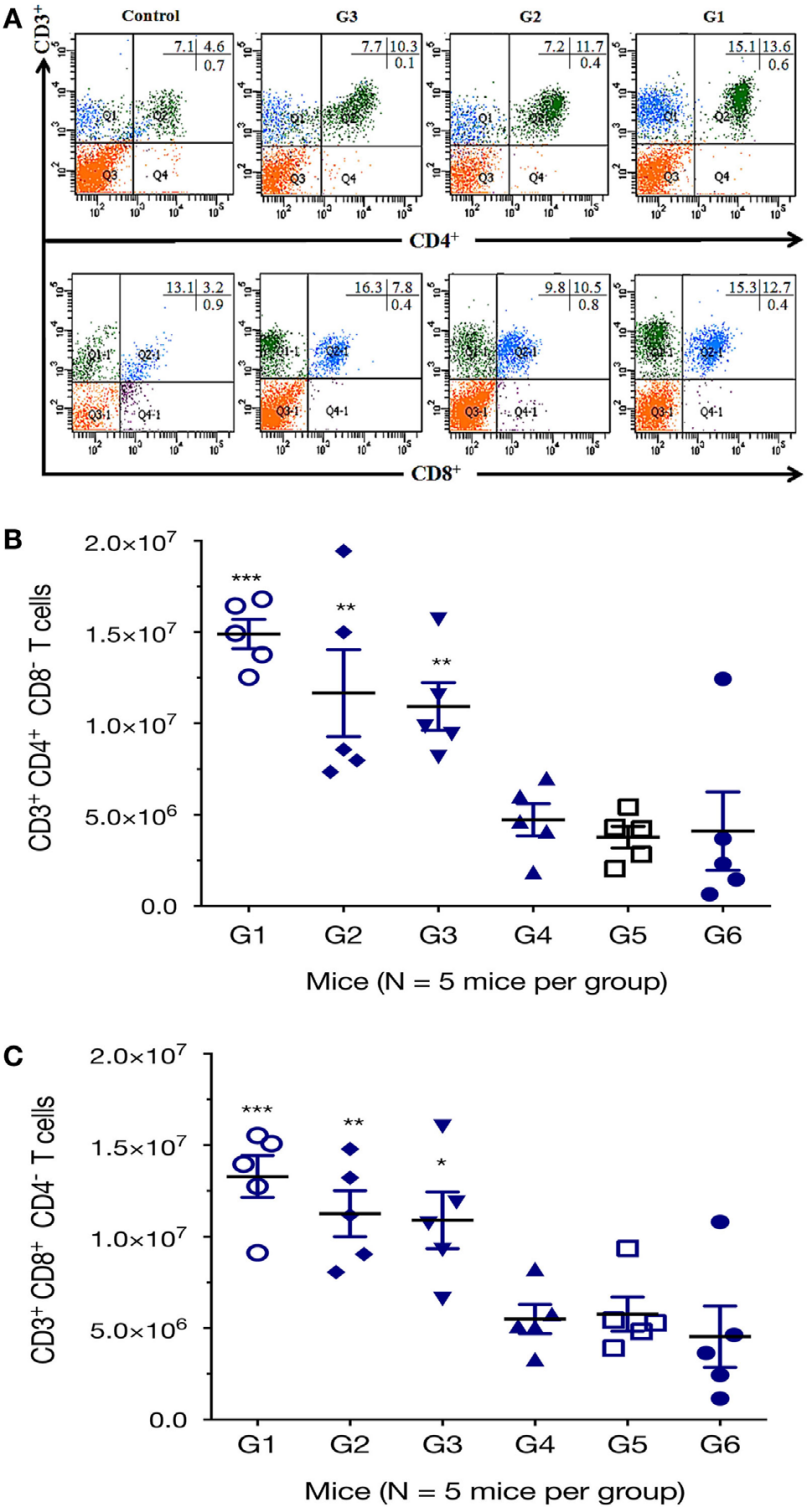
DNA immunization augmented the frequency of antigen-specific T cells. Percentages of CD4^+^ and CD8^+^ T cells subsets were determined in the spleen of mice 2 weeks after the final immunization by flow cytometry analysis. **(A)** Representative dot plots showing the percentages of CD3^+^ CD4^+^ CD8^−^ and CD3^+^ CD8^+^ CD4^−^ T lymphocytes. **(B)** Total numbers of CD3^+^ CD4^+^ CD8^−^ T lymphocytes per spleen. **(C)** Total numbers of CD3^+^ CD8^+^ CD4^−^ T lymphocytes per spleen. Experimental groups include G1: mice received pVAX1 plasmids containing five antigens (ROP16, ROP18, MIC6, CDPK3, and PF); G2: mice received pVAX1 plasmids containing four antigens (ROP16, ROP18, MIC6, and CDPK3); G3: mice received pVAX1-PF; G4: mice received an empty pVAX1; G5: mice received phosphate-buffered saline alone; G6: healthy control mice. Data are mean ± SDs (representative of three experiments). *p < 0.05, **p < 0.01, and ***p < 0.001, compared with the control groups.

We further characterized the cellular immune response, using flow cytometry analysis, and found that the numbers of CD3^+^ CD4^+^ CD8^−^ T cells in spleens of mice from G1, G2, and G3 were significantly higher than those from the controls (Figure 4). One-way ANOVA and Tukey’s multiple comparisons test of the absolute numbers of CD3^+^ CD4^+^ CD8^−^ T cells in spleens, showed that G1 versus G4 (*p* = 0.0010), G1 versus G5 (*p* = 0.0003), G1 versus G6 (*p* = 0.0005); G2 versus G4 (*p* = 0.0359), G2 versus G5 (*p* = 0.0130), G2 versus G6 (*p* = 0.0187); G3 versus G4 (*p* = 0.0752), G3 versus G5 (*p* = 0.0287), G3 versus G6 (*p* = 0.0407). After the third vaccination, mice immunized with various vaccines produced significantly higher CD3^+^ CD8^+^ CD4^−^ T cells in splenic lymphocytes as did the controls. The numbers of CD3^+^ CD8^+^ CD4^−^ T cells in mice from G1, G2, and G3 were significantly increased compared with those from control mice (Figure 4). One-way ANOVA and Tukey’s multiple comparisons test of the absolute numbers of CD3^+^ CD8^+^ CD4^−^ showed that G1 versus G4 (*p* = 0.0026), G1 versus G5 (*p* = 0.0038), G1 versus G6 (*p* = 0.0007); G2 versus G4 (*p* = 0.0381), G2 versus G5 (*p* = 0.0527), G2 versus G6 (*p* = 0.0111); G3 versus G4 (*p* = 0.0590), G3 versus G5 (*p* = 0.0804), G3 versus G6 (*p* = 0.0178). These findings suggest that the frequency of CD4^+^ CD8^+^ T cells were augmented after DNA immunization.

### Cytokine Production by Spleen Cells

**Figure 5:**
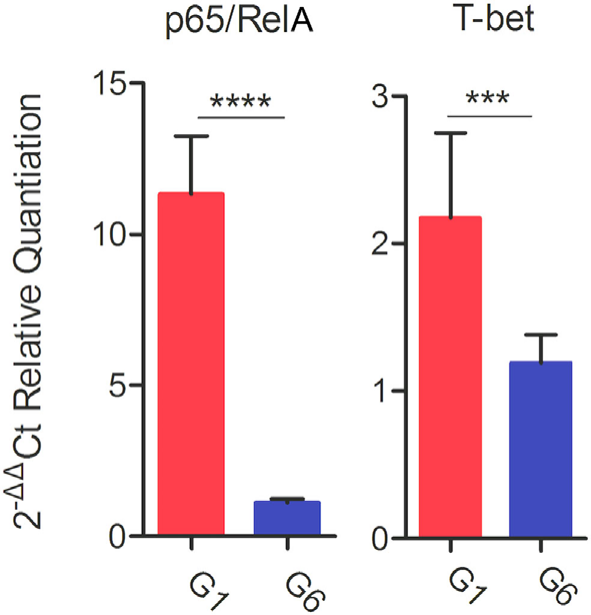
Real-time-PCR expression analysis of the transcription factors p65/RelA and T-bet. β-Actin was used as a housekeeping reference gene. Experimental groups include G1: mice received pVAX1 plasmids containing five antigens (ROP16, ROP18, MIC6, CDPK3, and PF); G6: healthy control mice. Each sample was analyzed in triplicate. Data are mean ± SDs (error bars) from the two mice. ***p < 0.001 and ****p < 0.0001.

Following stimulation with TLA, significantly high levels of IFN-γ, IL-2, and IL-12 were observed in splenocyte cultures from mice in G1, G2, and G3 when compared with those from control mouse groups (Table 3). In regard to IL-2, G1, G2, and G3 were all significantly higher than G4, G5, and G6 (all *p* < 0.0001). For IL-12, G1, G2, and G3 were all significantly higher than G4, G5, and G6 (all *p* < 0.0001), and for IFN-γ, G1, G2, and G3 are all significantly higher than G4, G5, and G6 (all *p* < 0.001). Levels of IL-4 in mice immunized with various DNA vaccines were not significantly different than those in mice from control groups (*p* = 0.5028). We analyzed the expression level of the transcription factors p65/RelA and T-bet using RT-PCR. We examined the difference between the expression of these two genes between control mice and mice in G1. Results showed that the expression of both p65/RelA and T-bet genes was significantly higher in G1 mice than in the control group (*p* < 0.0001 and *p* = 0.0010) (Figure 5). This result indicates that p65/RelA and T-bet are induced by immunization and are likely to increase the production of IFN-γ and IL-12 cytokines.

**Table 3:** Cytokine production by splenocytes of immunized Kunming mice after stimulation by *T. gondii* lysate antigen.

Group^b^	Cytokine production (pg/ml)^a^			
	
	Gamma-interferon	Interleukin-2 (IL-2)	IL-4	Interleukin-12 (IL-12)
G1	1,003.66 ± 311.02	294.24 ± 11.1	<10	411.96 ± 57.94
G2	644.94 ± 190	269.35 ± 19.17	11.1 ± 17.4	184.75 ± 39.95
G3	1,930.26 ± 5.46	277.14 ± 2.24	<10	400.14 ± 54.43
G4	169.29 ± 1.66	<50	<10	15.42 ± 2.01
G5	182.94 ± 33.64	<50	<10	<10
G6	177.75 ± 22.32	<50	<10	<10

*^a^No significant difference (*p* > 0.05) in the level of the four cytokines was observed between the immunized groups (G1, G2, and G3) nor between the control groups (G4, G5, and G6). Differences between immunized groups and control groups were significant for INF-γ (*p* < 0.001), and for IL-2 and IL-12 (*p* < 0.0001). Levels of IL-4 did not show any significant differences between the different mouse groups (*p* > 0.05)*.

*^b^Experimental groups included G1: mice received pVAX1 plasmids containing five antigens (ROP16, ROP18, MIC6, CDPK3, and PF); G2: mice received pVAX1 plasmids containing four antigens (ROP16, ROP18, MIC6, and CDPK3); G3: mice received pVAX1-PF; G4: mice received an empty pVAX1; G5: mice received phosphate-buffered saline alone; G6: healthy control mice. Data are mean ± SDs for three wells (representative of three independent experiments)*.

### Assessment of Protective Activity

**Figure 6:**
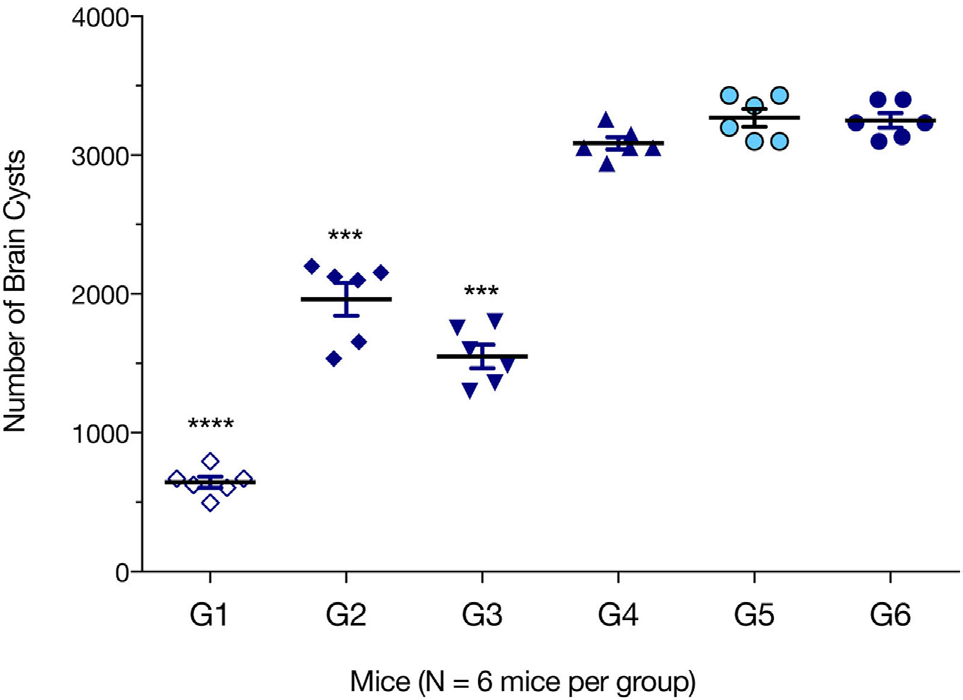
Protection against chronic toxoplasmosis in immunized mice 2 weeks after the final booster immunization. Six mice per group were challenged orally with a dose of 10 cysts of the Pru strain (type II). Cyst load was counted from whole brain homogenates of mice 6 weeks after challenge. Experimental groups include G1: mice received pVAX1 plasmids containing five antigens (ROP16, ROP18, MIC6, CDPK3, and PF); G2: mice received pVAX1 plasmids containing four antigens (ROP16, ROP18, MIC6, and CDPK3); G3: mice received pVAX1-PF; G4: mice received an empty pVAX1; G5: mice received phosphate-buffered saline alone; G6: healthy control mice. Data are mean ± SDs (representative of three experiments). ***p < 0.001 and ****p < 0.0001, compared with the control groups.

We evaluated which immunization regimen generated an immune response that was strong enough to protect against the formation of *T. gondii* brain cysts. Six mice from each group were challenged with 10 cysts of *T. gondii* Pru strain; and brain cyst loads were determined 6 weeks later. As shown in Figure 6, mice from G1, G2, and G3 had significantly lower numbers of brain cysts than those from control groups G4, G5, and G6 (all *p* < 0.0001). The lowest number of brain cysts was detected in immunized mice from G1 (643.33 ± 89.63), which represented a significant reduction (80.22%, *p* < 0.0001) when compared with the number of cysts found in control non-immunized + challenged mice (3,244.33 ± 96.42). The number of brain cysts in G1 mice was significantly lower than in G2 or G3 mice (*p* < 0.0001).

## Discussion

Despite significant research to develop and evaluate anti-*T. gondii* vaccines, there is little consensus on the “best” antigens to target and the optimal means of targeting them. The development of vaccines to prevent cerebral toxoplasmosis disease in high risk populations could reduce the enormous tragedy associated with brain infection with cystogenic (brain cyst-forming) strains of *T. gondii*. A substantially reduced parasite cyst load in the brains of immunized mice in our study, together with enhanced humoral and cell-mediated immune responses—compared with non-immunized infected mice demonstrate that immunization with a DNA cocktail vaccine of five antigens, provides considerable protection in mice against a primary oral challenge with the avirulent cystogenic *T. gondii* Pru strain. These elements are believed to be important in developing an effective therapeutic vaccine. Our results showed that immunization using the cocktail DNA vaccine is a promising approach to control chronic toxoplasmosis, when compared with vaccination based on single antigens (31–35). In this study, cocktail DNA vaccine achieved an 80.22% reduction in the parasite cyst burden, compared with 50% obtained by DNA vaccine expressing *T. gondii* CDPK3 (34) and 39.08% by DNA vaccine (pVAX1-PF) encoding TgPF gene (35). Reduction in the brain cysts’ load achieved in our study, was also greater than the 57.8% reduction achieved by vaccination using multiple antigenic peptides encapsulated by chitosan microspheres (36).

The cytokine Th1 immune response is crucial for the control of infection with this obligate intracellular pathogen (14, 37). In our study, immunized mice developed a high level of anti-*T. gondii* IgG antibodies, particularly after the third booster immunization. It is arguable that a high level of antibodies plays an important role in protection against subsequent infection with *T. gondii* tachyzoites, and in controlling *T. gondii* during chronic infection, by preventing cysts’ reactivation. A requirement for B cells, in addition to cell-mediated immunity, has been reported for mice challenged with virulent *T. gondii* parasites after vaccination with attenuated tachyzoites—suggesting that antibody-mediated immunity is also critical for *T. gondii*-induced protection (38). Our previous studies have demonstrated that the individual protective immunity offered by pVAX1-PF, pVAX1-ROP16, pVAX1-ROP18, pVAX1-MIC6, or pVAX1-CDPK3 against *T. gondii* infection, elicited a mixed Th1/Th2 or Th1-biased immunity *via* the induction of lymphocyte proliferation, activation of CD8^+^ T cells and increased IFN-γ production (31–35). In this study, a mixed Th1/Th2 immune response, with a slight bias toward the Th1-type response, was detected in mice in groups G1, G2, and G3, as indicated by a slightly increased lgG2a/IgG1 ratio (i.e., a higher level of IgG2a than IgG1). Vaccination using five antigens evoked an increase in CD8^+^ T lymphocytes and IFN-γ production along with low levels of IL-4 in G1 mice compared with controls; all are characteristic features of a Th1-type cellular immune response. These findings indicate that a mixed Th1/Th2 protective immune response was elicited following immunization with the cocktail vaccine—consistent with the results of previous studies (14, 39).

A cellular immune response, including high levels of IFN-γ, CD4^+^, and CD8^+^ T lymphocytes, is required for protection against chronic *T. gondii* infection (39–43). Both cell types act synergistically to control *T. gondii* infection, and effective control of *T. gondii* infection requires CD4^+^ for the generation of proficient CD8^+^-mediated immunity (44). CD8^+^ T cells possess anti-cyst activity, mediated by a perforin-dependent mechanism (45, 46) and high levels of CD8^+^ T lymphocytes can contribute to a decrease of brain cyst loads (39, 43, 47–50). In our study, the significantly increased numbers of CD8^+^ T cells in mice immunized with our cocktail vaccine may have contributed toward reducing the brain cyst burden.

Gamma-interferon, a crucial mediator of immune resistance to *T. gondii* infection, was detected in elevated levels in mice from G1, G2, and G3. IFN-γ can activate macrophages and the CTL response during infection (51). Significantly higher expression of the transcription factor T-bet (*p* = 0.0010) was observed in mice in G1, compared with mice from the control group. This finding indicates that increased IFN-γ production may have resulted from T-bet-mediated activation of CD4^+^ T cells and natural killer (NK) cells (36). In marked contrast with IFN-γ, the level of IL-4 was not statistically different between any mouse groups. IL-4 is a Th2-type cytokine, produced in response to receptor activation by Th2-type CD4^+^ T cells, basophils, and mast cells, and possesses B-cell stimulatory and Th2-promoting properties (52). IL-4 functions are generally antagonistic to those of IFN-γ, in line with the increased IFN-γ and decreased IL-4 levels seen in our study. Elevated IFN-γ production, together with a low level of IL-4, was also detected in mice immunized with DNA vaccines encoding TgROP1 (15), TgROM5 (39), TgCDPK2 (53), TgSAG1 (54), or multi-antigens (17, 19). IL-4 also promotes isotype-switching in murine B cells to IgG1 and IgE, but inhibits switching to IgG2a, IgG2b, and IgG3. This is in agreement with the reduced IL-4 and a high IgG2a to IgG1 ratio, observed in our study—providing more evidence of a biased Th1 type immune response.

Previous studies have shown that high levels of CD4^+^ and IL-2 production, increased mouse resistance to chronic toxoplasmosis (30, 41, 42, 55). We also found an increased production of CD4^+^, IL-2, and IL-12, along with a reduction in the brain cyst load in mice vaccinated with various DNA vaccines. A major role of NF-κB in resistance to *T. gondii* is the induction of IL-12 secretion (56). IL-12, putatively *via* STAT4, is important for the optimal production of INF-γ, which in turn induces differentiation of Th1 T lymphocytes, and possibly CD8^+^ and NK cells, to control *T. gondii* infection. Significantly increased expression of the transcription factor p65/RelA (a transcription factor of the NF-κB pathway) was observed in mice in G1, when compared with mice in the control group (*p* < 0.0001). This result, in addition to the increased level of IFN-γ, presents activation of NF-κB pathway as an additional mechanism for increased IFN-γ production to limit *T. gondii* infection. Suppressed expression of IL-12 resulted in 100% mortality in mice infected with *T. gondii* (57). High levels of IL-12 contributed to brain cyst reduction in mice (54, 58). Toll-like receptor 11 (TLR11) signaling is an important pathway involved in the production of IL-12 (23, 59). *T. gondii* profilin (TgPF) can act as a ligand for TLR11 to mediate cytokine production (60–62) and has been exploited as a TLR-based vaccine adjuvant to enhance immune responses generated by vaccination (62–65). TgPF also plays an essential role in *T. gondii* gliding motility, invasion and egress from host cells (23, 24) and is an immunodominant antigen (35, 66). These key characteristics of TgPF prompted us to separately evaluate the protective efficacy of immunization with this gene against chronic toxoplasmosis. The specific finding that DNA vaccine encoding TgPF alone induced the production of high level of IL-12 is consistent with previous data (60) and, together with a higher IgG2a/IgG1 ratio and reduced brain cyst numbers may be of particular relevance. The enhanced protective immunity seen in mice from G1, compared with mice from group G2, suggests that TgPF may be necessary to augment the immune response induced by the multicomponent *T. gondii* DNA vaccine used in our study.

## Conclusion

We have demonstrated that a strong humoral and cellular immune response, conferring significant protection against chronic *T. gondii* infection in mice—after immunization three times at 2-week intervals with a DNA vaccine encoding multiple antigens (TgPF, TgROP16, TgROP18, TgMIC6, and TgCDPK3). Several DNA immunizations were necessary to elicit the specific IgG antibody response. Immunization with several plasmids expressing more antigens produced a greater antibody response when compared with immunization using fewer plasmids expressing less number of antigens. These data support a call for further evaluation of multivalent synthetic plasmids as potential therapeutic *T. gondii* vaccines. Our findings are significant as they open up the possibility that chronic toxoplasmosis can be controlled by the combined action of multiple parasite-derived antigens. Although further studies and clinical evaluations are required, this study puts into place a “proof of concept” that tests the efficacy of the combination of a range of *T. gondii* antigens in preventing a chronic brain infection that is incurable with current therapeutics.

## Ethics Statement

All animal protocols were reviewed and approved by the Animal Administration and Ethics Committee of Lanzhou Veterinary Research Institute, Chinese Academy of Agricultural Sciences. The study was performed in strict compliance with the recommendations set forth in the Animal Ethics Procedures and Guidelines of the People’s Republic of China. All efforts were made to minimize animal suffering and to reduce the numbers of animals used in the experiments.

## Author Contributions

X-QZ, N-ZZ, and HE designed the experiments, interpreted the data, and critically revised the manuscript. N-ZZ, QG, MW, BW, and J-LW performed the experiments and analyzed the data. N-ZZ drafted the manuscript. F-KZ, MW, and L-YH helped in the implementation of the study. All the authors reviewed and approved the final version of the manuscript.

## Conflict of Interest Statement

The authors declare that the research was conducted in the absence of any commercial or financial relationships that could be construed as a potential conflict of interest.
